# Efficacy and Mechanisms of Gastric Volume-Restriction Bariatric Devices

**DOI:** 10.3389/fphys.2021.761481

**Published:** 2021-10-28

**Authors:** Yanmin Wang, Ghassan S. Kassab

**Affiliations:** California Medical Innovations Institute, San Diego, CA, United States

**Keywords:** obesity, weight loss, medical device, restrictive procedure, review

## Abstract

Obesity is a chronic disease that affects over 795 million people worldwide. Bariatric surgery is an effective therapy to combat the epidemic of clinically severe obesity, but it is only performed in a very small proportion of patients because of the limited surgical indications, the irreversibility of the procedure, and the potential postoperative complications. As an alternative to bariatric surgery, numerous medical devices have been developed for the treatment of morbid obesity and obesity-related disorders. Most devices target restriction of the stomach, but the mechanism of action is likely more than just mechanical restriction. The objective of this review is to integrate the underlying mechanisms of gastric restrictive bariatric devices in obesity and comorbidities. We call attention to the need for future studies on potential mechanisms to shed light on how current gastric volume-restriction bariatric devices function and how future devices and treatments can be further improved to combat the epidemic of obesity.

## Introduction

Obesity is a consequence of caloric imbalance and excessive fat accumulation. The World Health Organization (WHO) defined obesity as body mass index (BMI) over 30, while 25–30 is considered overweight. Obesity is a major public health problem in the developed world, which significantly increases the risk of multiple diseases and disorders such as type 2 diabetes mellitus, hypertension, heart disease, and cancer. The prevalence of obesity has greatly increased in the past decades. It was estimated that in 2016, the number of children/adolescents and adults that suffered from obesity worldwide were 124 and 671 million, respectively (Bentham et al., 2017). In addition, 213 million children/adolescents and 1.3 billion adults were in the range of overweight (Bentham et al., 2017). In the US, the prevalence of obesity in adults and children ages 6–11 old has reached over 35% (Flegal et al., 2012) and 17% (Ogden et al., 2016).

In various countries and regions, bariatric surgery has been listed in obesity management guidelines as the most effective way to treat morbid obesity and the related disorders (Jensen et al., 2014; Yumuk et al., 2015; Wharton et al., 2020). The most popular procedures (American Society for Metabolic and Bariatric Surgery, 2021) gastric bypass and sleeve gastrectomy are, however, not readily accepted by many patients because both include removal of some part of the stomach, and this gastrectomy may induce severe complications. Only 1–2% of the eligible candidates undergo bariatric surgery for obesity each year in the US (Gasoyan et al., 2019). Furthermore, based on Western guidelines, patients whose BMI is lower than 35 (or 40 without adiposity-related disease) are beyond the indications of bariatric surgery and thus lack effective treatments.

As less invasive alternatives, many gastric restrictive bariatric devices such as gastric band, intragastric balloons, and so on, have been used for combating obesity and some achieve comparable efficacy to surgeries (Vargas et al., 2018). Although most of the devices are intended to restrict the stomach to decrease calorie intake, the mechanisms of action for the considerable weight loss following gastric volume-restricted bariatric devices are not fully appreciated. This review aims to integrate the potential mechanisms through which restrictive bariatric devices induce weight loss and metabolic improvements. To the best of our knowledge, this is the first review on this topic.

### Gastric Band

**FIGURE 1:**
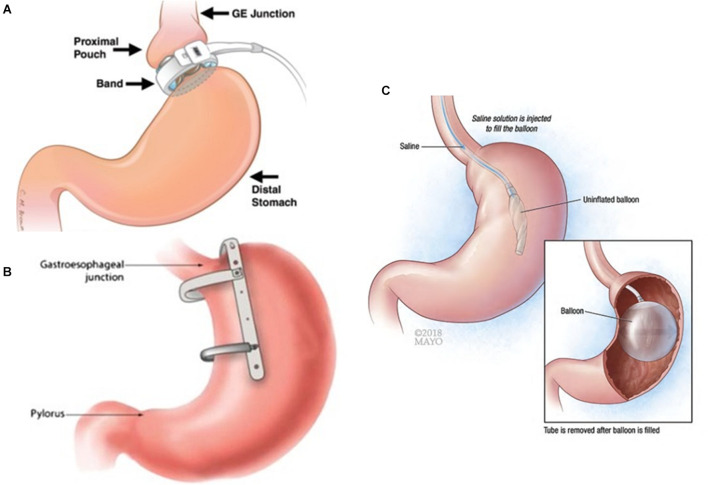
Schematic of gastric restrictive bariatric devices. **(A)** Adjustable gastric banding (AGB). Used with permission of the Radiological Society of North America (RSNA^®^) (Sonavane et al., 2012). The band is planted around the stomach below gastroesophageal (GE) junction. **(B)** Gastric sleeve implant (GSI). Reprinted by permission from Springer Nature, *Obesity Surgery*, Efficacy of a Laparoscopic Gastric Restrictive Device in an Obese Canine Model, Guo et al. (2014) COPYRIGHT 2013. The device is mounted on the lesser curvature and creates a vertical sleeve food track. **(C)** Intragastric balloons (IGB). Used with permission of Mayo Foundation for Medical Education and Research, all rights reserved (https://www.mayoclinic.org/medical-professionals/endocrinology/news/intragastric-balloon-a-re-emerging-approach-for-obesity/mac-20430245). The inflated balloon occupies some intragastric space.

In the adjustable gastric banding (AGB) procedure, an adjustable silicone band is placed around the stomach below the gastro esophageal junction to restrict the dilation of the gastric pouch as shown in Figure 1A. AGB is the most well-known gastric restrictive device: first implanted in 1983 (Kuzmak, 1991), it gained popularity in early twenty-first century (Favretti et al., 2009; Ibrahim et al., 2017). A meta-analysis (Garb et al., 2009) found that the excess weight loss (weight loss/pre-operative excess body weight × 100%) post-AGB was 42.6% at 1 year, 50.3% at 2 years, and 55.2% at over 3 years. Another meta-analysis (Golzarand et al., 2017) showed that AGB induced nearly 48% excess weight loss at either 5 or 10 years postoperatively. According to data from 20 years follow-up in patients with obesity, AGB was associated with significantly lower incidence of diabetes, cardiovascular diseases, cancer, and renal diseases (Pontiroli et al., 2018). The cost for AGB is significantly lower than that for Roux-en-Y gastric bypass or sleeve gastrectomy (SG) (Doble et al., 2019). Some studies, however, reported that AGB failed to maintain reduced body weight or control obesity-related morbidities (Pournaras et al., 2010; Chang et al., 2014; Park et al., 2019). Worse still, additional studies showed that patients who underwent AGB may need a second surgery due to band migration or erosion, pouch dilatation, achalasia or megaesophagus, stomach obstruction, or other severe complications (Arias et al., 2009; Chang et al., 2014; Kodner and Hartman, 2014; Tsai et al., 2019). The reported reoperation rate was up to 82.7% in 15-year follow-up (Tsai et al., 2019). As a result, the popularity of AGB has been dramatically decreased in the past decade. In recent years, several improved AGB devices and systems (Billy et al., 2014; Edelman et al., 2014; Ponce et al., 2014) have been developed, but the long-term effects remain unclear. In 2019, AGB only accounted for 0.9% of bariatric procedures in the US (American Society for Metabolic and Bariatric Surgery, 2021).

There have been numerous studies focused on the potential mechanism of AGB in weight control and metabolic amelioration induced by the placement of the band. AGB is considered to improve eating behavior such as alleviating binge eating disorders and decreasing emotional eating and night eating in the short term (Opozda et al., 2016; Hindle et al., 2020), whereas long-term results are inconsistent (Opozda et al., 2016; Smith et al., 2019). Monteiro et al. (2007) compared AGB rats and pair-fed rats, observing that AGB rats were leaner. This study suggests that additional factors beyond restriction exist. It seems that gastric motility, neural activity, ghrelin level, concentrations of gut hormones, energy expenditure, bile acids metabolism, and gut microbial diversity play important roles; however, the conclusions varied significantly (Wang et al., 2019). For example, Aron-Wisnewsky et al. (2019) observed that gut microbial gene abundance increased after AGB whereas Lee et al. (2019) reported an opposite result. Another example is that ghrelin levels were found to be unchanged (Sysko et al., 2013), increased (Kawasaki et al., 2015), or decreased (Leonetti et al., 2003) following AGB. We assume that the variations are not only partly due to the differences in techniques of the procedures and baseline conditions of the subjects, but also because the underlying factors are complex (i.e., multiple mediators work together and interact with each other).

In addition to AGB in which the stomach is restricted horizontally, vertical banded gastroplasty (VBG) used banding above the crow’s foot of Latarjet’s nerve along with vertical staple line toward the angle of His to restrict the stomach. In the early 1980s, Mason (1982) reported that VBG caused more weight loss and less complications when compared with other surgical procedures. Kellum et al. (1990) reported that at 6 months after VBG, excess weight loss in patients with morbid obesity was 41.8%. Brolin et al. (1994) found that patients underwent VBG preferred to eat high-caloric food, resulting in postoperative weight regain. Olbers et al. (2006) obtained similar results, showing that VBG patients consumed more sweet foods and less vegetables and fruits. One study (Amsalem et al., 2014) revealed that VGB (specifically the silastic ring vertical gastroplasty) as well as AGB significantly lower the risk of pregnancy complications such as gestational diabetes mellitus and hypertension. This suggests that some metabolic factors exist in these restrictive procedures, which requires further research. In Kellum et al. (1990)’s study, glucose, insulin, enteroglucagon, serotonin, vasoactive intestinal polypeptide, and cholecystokinin (CCK) responses to meals were not changed after VBG. Tremaroli et al. (2015) suggested that VBG has long-term positive effects on gut microbiota and bile acids. The resting energy expenditure was reported to be decreased after VBG, but it seemed a reflection of weight loss instead of the reason (Olbers et al., 2006). Similar to AGB, however, long-term studies (Balsiger et al., 2000; van Wezenbeek et al., 2015; Froylich et al., 2020) revealed that the weight reduction after VBG was not sustained and complications such as pouch dilatation, staple-line disruptions, and outlet stenosis were frequent. Therefore, VBG lost popularity and is no longer practiced.

### Gastric Sleeve Implant and Gastric Clip

Since so-called restrictive procedures are technically simple, there have been several devices designed to treat obesity by reducing gastric volume, apart from traditional gastric banding devices, in either laboratory or clinical settings. Our group developed a restrictive device (referred to as Gastric Sleeve Implant, GSI), which is designed to be laparoscopically implantable and removable (Guo et al., 2011, 2014) as shown in Figure 1B. When placed loosely on the outside (serosa) of the stomach, the device generates a sleeve-shaped pouch similar to sleeve gastrectomy (SG) but avoids the irreversibility of the SG because it does not require stapling or gastrectomy. When the stomach is empty, GSI does not compress the stomach, which reduces the risk of device migration or tissue necrosis. GSI also has two C-rings to prevent the distension of the sleeve (Guo et al., 2011, 2014). The GSI is safe, effective and has been proven removable in animals (Guo et al., 2011, 2014). In a canine model, the excess weight loss reached 75% at 12 weeks after procedure but returned to 22% at 6 months after the removal of the device (Guo et al., 2014). To explore the underlying mechanism, our canine and rat studies (Guo et al., 2012) showed an elevated level of ghrelin and a reduced concentration of leptin after the implantation of GSI, which returned to normal levels after GSI removal. We assume that GSI induces an adaptive or compensatory increase in ghrelin secretion at early stages after surgery due to the integrity of stomach, which would counteract additional weight loss and cause the corresponding body weight recovery after its removal (Guo et al., 2012). The lower leptin level induced by GSI is highly correlated with weight loss. It is probably secondary to weight loss as serum leptin concentration reflects the total amount of fat mass in the body (Guo et al., 2012). The Glucagon-like peptide-1 (GLP-1) concentration was found to be unchanged.

Subsequently, a device with similar principle, the vertical gastric clip (Jacobs et al., 2017; Noel et al., 2018) or BariClip (Noel et al., 2020), was used in patients. Parallel to the lesser curvature, the device separates a medial lumen from an excluded lateral gastric pouch (Jacobs et al., 2017). The reduction of BMI and % excess weight loss were 12.7 and 66.7, respectively, at 2 years after the operation (Jacobs et al., 2017). In addition, the quality of life was improved in more than 90% of patients (Noel et al., 2018). A simpler device named Gastric Clip (Chao et al., 2019) was also used in clinics. The gastric clip creates a transverse gastric partition when obliquely applied to the upper fundus (Chao et al., 2019). One year after surgery, BMI was significantly reduced from 44 to 37 kg/m^2^, and the total weight loss (weight loss/pre-operative body weight × 100%) was 23.5%. Diabetes and hyperlipidemia were effectively alleviated as well (Chao et al., 2019), and the effects were much better when combined with a proximal jejunal bypass. The possible mechanisms underlying clip-induced weight change require further studies. The long-term benefits of these devices are currently lacking, however, and some patients were reported to suffer from gastric obstruction or insufficient weight loss after such procedures and thus underwent clip removal or revisional surgery (de la Plaza Llamas et al., 2020; Chang et al., 2021). Furthermore, gastric clip has been used to assist with SG, but a gastrectomy was still performed to achieve metabolic improvements in mice (Schlager et al., 2011; Wei et al., 2020). This implies that a simple gastric clip may not be a reliable bariatric device as a stand-alone. More follow-up data is needed.

### Intragastric Balloons

Intragastric balloons (IGB) have been used to occupy the gastric space by endoscopic placement as shown in Figure 1C. The FDA has approved three IGBs (Vyas et al., 2017; Vargas et al., 2018), i.e., Orbera, Obalon, and ReShape Duo [no longer available (FDA, 2020)] to combat obesity with BMI 30–40 kg/m^2^. In addition, there have been some other IGBs (such as Elipse, Medsil, Spatz3, and so on) (Bužga et al., 2014; Ramai et al., 2020; Badurdeen et al., 2021) awaiting for validation or approval. As a result, reducing gastric capacity via endoscopically implanted IGBs has emerged as a viable option to alleviate morbid obesity. A retrospective study of 5,874 patients (Fittipaldi-Fernandez et al., 2020) showed that the incidence of gastric perforation and digestive bleeding were only 0.07 and 0.05% in the first half year after IGB implant. According to the American Society for Metabolic and Bariatric Surgery (2021), in 2015, balloons were used only in 0.3% cases of bariatric procedures in the US, while in 2019 the number increased to 1.8%.

Generally, the balloons are placed endoscopically into stomach for no more than 6 months (in some techniques the time is longer), after which they are removed. A meta-analysis including 5,668 subjects (Popov et al., 2017) reported that patients had 28% excess weight loss and 4.8 kg/m^2^ BMI decrease at 6 months after IGBs removal, although some weight regain was observed at balloon removal. Some investigators showed that after 6-month implantation, the total weight loss of the IGBs is 6.8–13.2% (Vargas et al., 2018); at 12 months, i.e., 6 months following balloon removal, the weight loss is still satisfactory, albeit modest at 7.6–11.3% total weight loss (Vargas et al., 2018). This indicates that the weight reduction outcome after IGBs is not dependent on gastric restriction. Genco et al. (2013) reported that IGB placement improves eating habits, reducing frequent food consuming, preference of sweet foods, emotional eating, and after-dinner grazing in patients with obesity. Some IGBs are reported to alter gastric motility and hormone levels in addition to reducing gastric volume. Mion et al. (2005) found that balloon placement leads to suppression of gastric emptying and ghrelin production, but the subsequent weight reduction is not associated with gastric emptying. Another study (Mathus-Vliegen and de Groot, 2013) reported a decrease of CCK after IGBs, which may correlate with delayed gastric emptying. In these studies, the variations of ghrelin and CCK are likely to be the results of weight loss rather than the causes. Fuller’s group (Fuller et al., 2013) performed a randomized controlled trial for IGB evaluation. In their study, ghrelin was increased and leptin was decreased when the device was implanted, but both concentrations recovered to baseline after the removal of the balloon. In addition, fasting levels of adiponectin or Peptide YY (PYY) were not affected by weight loss associated with the IGBs treatment. Similarly, Bužga et al. (2014) observed that serum ghrelin was increased while leptin and fibroblast growth factor 21 levels were decreased at 6 months after balloon insertion in patients with BMI of 43 kg/m^2^, but longer-term results were not assessed. Another study (Mathus-Vliegen and Eichenberger, 2014) also indicated that fasting and postprandial ghrelin levels remained stable at 13 and 26 weeks after IGBs, despite sustained weight loss. A study (Badurdeen et al., 2021) showed that 9-month administration of Liraglutide (GLP-1 agonist) after IGB removal was effective in preventing weight regain and reducing fat mass. It indicates that GLP-1 concentration is potentially an important factor of IGB-induced weight loss, which needs further verification.

Aside from the potential changes in gastrointestinal motility and hormones, IGB therapy reduces fat mass and resting metabolic rate (Gaździńska et al., 2020), which are associated with weight decrease. IGBs are also reported to improve obesity related disorders such as hypertension (Popov et al., 2017), hyperglycemia (Popov et al., 2017), dyslipidemia (Ramai et al., 2020), and non-alcoholic fatty liver disease (Chandan et al., 2021). More studies are needed to reveal deeper mechanisms.

### Endoscopic Gastroplasty and Gastroplication

Endoscopic Sleeve Gastroplasty (ESG) is also an emerging endoluminal method to manage obesity. First used in patients a decade ago, it has been improved in recent years (Kumar et al., 2018). Using endoscopic suturing devices, ESG procedure places a series of sutures from the antrum to the fundus, creating a banana-shape stomach pouch like SG does. Similar devices to mimic SG or gastroplasty include Apollo OverStitch, EndoCinch, Incisionless Operating Platform, amongst others (Kumar, 2015).

In comparison of laparoscopic SG and AGB, although SG achieved the greatest weight reduction, ESG is thought to be the safest and most viable choice with lower morbidity and shorter stay in hospital (Novikov et al., 2018). Jain et al. (2017) summarized nine single center prospective human studies treating obesity by ESG technique. In these studies, no intra-procedure complication was reported, while 2.3% of the patients experienced major but not fatal postoperative complications such as perigastric leakage. Although the detailed techniques were different, the % excess weight loss was reported to be 30–57 (Jain et al., 2017). An international multicenter study (Barrichello et al., 2019) showed that at 12-month after ESG, the total and excess weight loss were 15.1 and 59.4%, and adipose tissue was significantly lowered. Lopez-Nava and coworkers retrospectively analyzed 248 patients, indicating that at 6 and 24 months after ESG, the total weight loss was 15.2 and 18.6%, respectively (Lopez-Nava et al., 2017). In another study with a smaller group of patients, they found that at 1 year after ESG, BMI loss was 7.3 kg/m^2^, while total and excess weight loss were 18.7 and 54.6% (Lopez-Nava et al., 2016). Alqahtani et al. (2019) provided similar data, showing 13.7, 15, and 14.8% total weight loss at 6, 12, 18 months, respectively. In this study, ESG also resulted in satisfactory remissions of diabetes, hypertension, and dyslipidemia (Alqahtani et al., 2019). Sharaiha et al. (2017) studied 91 consecutive patients who underwent ESG. At 1 year after procedure, the patients not only lost 14.4% body weight, but also showed significantly improved levels of hemoglobin A1c, systolic blood pressure, alanine aminotransferase, and serum triglycerides (Sharaiha et al., 2017).

There have been some studies exploring the underlying contributors of weight loss and metabolic improvements beyond restriction following ESG. Lopez-Nava et al. (2020) found a reduced levels of leptin and an improved insulin secretory pattern in patients at 6 months after ESG, while ghrelin, GLP-1, PYY, and adiponectin remained stable. These changes differed from those following SG, which is likely because of the different post-operatively anatomical structures between the two procedures. The researchers concluded that hormonal variations play little role in weight loss and metabolic improvements (Lopez-Nava et al., 2020). In contrast, Abu Dayyeh et al. (2017) revealed that insulin sensitivity was improved after ESG, with decreased (not significantly) ghrelin levels and unchanged leptin, GLP-1, and PYY. Moreover, they reported that ESG delays gastric emptying, thus producing early satiation and decreasing caloric consumption to reach maximum fullness in patients, but the sample size was to be increased (Abu Dayyeh et al., 2017). This finding is in support of the above-mentioned Lopez-Nava et al.’s conclusion, although the gut hormone changes in the two papers were not comparable. The variation may mainly be due to different follow-up duration as well as baseline conditions of the subjects.

The articulating circular endoscopic (ACE) stapler is a transoral bariatric device for endoscopic gastroplication which has identical principle to ESG. Paulus et al. (2020) reported that in subjects whose BMI was 38.3 kg/m^2^ at baseline, BMI decreased to 33.9 kg/m^2^ at 1 year postoperatively. After the procedure, patients had a downregulated ghrelin gene expression as well as its activating enzyme in the upper gastrointestinal tract and increased level of plasma adiponectin (van der Wielen et al., 2017). Trans-oral endoscopic restrictive implant system (De Jong et al., 2010; Verlaan et al., 2016) is another similar device. At 6 months after using the device, total and excess weight loss were 15.1 and 30.1%, but the longer-term effects were not reported yet and the biological mediators were to be explored.

### Other Bariatric Technologies

It should be noted that there are other bariatric devices than we could include in the rapidly developing field, and every technique has both the pros and cons. Our current review mainly focuses on mechanisms behind the gastric volume restricted devices. Understanding the possible mechanisms beyond restriction will help us better understand the pathophysiology of obesity and provide the potential to develop more effective approaches to combat the epidemic of obesity. Table 1 summarizes the factors that may contribute to weight control with device implants.

**TABLE 1:** Parameter changes after gastric volume-restriction bariatric devices.

	Adjustable gastric banding (AGB)	Vertical banded gastroplasty (VBG)	Gastric sleeve implant (GSI)	Intragastric balloons (IGB)	Endoscopic sleeve gastroplasty (ESG)	Articulating circular endoscopic (ACE)
Gastric emptying	↔↓			↓	↓	
Energy expenditure	↑↔↓	↓		↓		
Ghrelin level	↑↔↓		↑	↑↔↓	↔↓	↓
Glucagon-like peptide 1 level	↔↓		↔		↔	
Peptide YY level	↑↔			↔	↔	
Cholecystokinin level		↔		↓		
Leptin level			↓	↓	↔↓	
Adiponectin level				↔	↔	↑
Bile acids	↑↓	↑				
Gut microbiota	Gene richness ↑↓; Proteobacteria ↑	E. Coli ↑; Eubacterium rectale ↓; Roseburia intestinalis ↓				
Eating habit	Binge eating ↔↓; emotional eating ↔↓; night eating ↓; grazing ↑	High-caloric food ↑; sweet food ↑; vegetable↓; fruit ↓		Grazing ↓; emotional eating ↓; sweet food ↓; after-dinner grazing ↓		

*↑, increased; ↔, unchanged; ↓, decreased. More than one arrow indicates inconsistent data; blank means unknown data.*

## Conclusion

Although many gastric volume-restriction bariatric devices have been developed for laboratory or clinical use, the underlying mechanism of the devices in alleviating morbid obesity and comorbidities is still not fully understood. Despite the fact that the “restrictive” devices physically limit or reduce gastric capacity, mechanical restriction may not have the key role in achieving the beneficial outcomes. Gastric motility and hormone responses may also contribute to the efficacy of the procedures. Changes in hormone levels provide some indication as to how these bariatric devices work; however, they do not necessarily provide a mechanism for the weight loss effects. Instead, these changes could be compensatory, rather than mediators. Further studies are required to determine whether these changes in hormone levels are in fact causal to weight loss. Studies regarding other factors that contribute to bariatric surgeries (Madsbad et al., 2014; Wang et al., 2019) such as vagal and hypothalamic activity, role of bile acids, and gut flora alterations are lacking. More studies are encouraged to elucidate the detailed mechanisms of weight and energy regulation and glucose metabolism after use of gastric bariatric devices.

## Author Contributions

YW searched and arranged literatures. GK engaged in the conception, design, and coordination of the work. Both authors participated in drafting and revising the manuscript.

## Conflict of Interest

The authors declare that the research was conducted in the absence of any commercial or financial relationships that could be construed as a potential conflict of interest.

## Publisher’s Note

All claims expressed in this article are solely those of the authors and do not necessarily represent those of their affiliated organizations, or those of the publisher, the editors and the reviewers. Any product that may be evaluated in this article, or claim that may be made by its manufacturer, is not guaranteed or endorsed by the publisher.

## References

[B1] Abu Dayyeh B. K. Acosta A. Camilleri M. Mundi M. S. Rajan E. Topazian M. D. (2017). Endoscopic sleeve gastroplasty alters gastric physiology and induces loss of body weight in obese individuals. *Clin. Gastroenterol. Hepatol.* 15 37–43.e1. 10.1016/j.cgh.2015.12.030 26748219

[B2] Alqahtani A. Al-Darwish A. Mahmoud A. E. Alqahtani Y. A. Elahmedi M. (2019). Short-term outcomes of endoscopic sleeve gastroplasty in 1000 consecutive patients. *Gastrointest. Endosc.* 89 1132–1138. 10.1016/j.gie.2018.12.012 30578757

[B3] American Society for Metabolic and Bariatric Surgery (2021). *Estimate of Bariatric Surgery Numbers, 2011-2019.* Available Online at: https://asmbs.org/resources/estimate-of-bariatric-surgery-numbers (accessed March 30, 2021).

[B4] Amsalem D. Aricha-Tamir B. Levi I. Shai D. Sheiner E. (2014). Obstetric outcomes after restrictive bariatric surgery: what happens after 2 consecutive pregnancies? *Surg. Obes. Relat. Dis.* 10 445–449. 10.1016/j.soard.2013.08.016 24342035

[B5] Arias I. E. Radulescu M. Stiegeler R. Singh J. P. Martinez P. Ramirez A. (2009). Diagnosis and treatment of megaesophagus after adjustable gastric banding for morbid obesity. *Surg. Obes. Relat. Dis.* 5 156–159. 10.1016/j.soard.2008.11.007 19250879

[B6] Aron-Wisnewsky J. Prifti E. Belda E. Ichou F. Kayser B. D. Dao M. C. (2019). Major microbiota dysbiosis in severe obesity: fate after bariatric surgery. *Gut* 68 70–82. 10.1136/gutjnl-2018-316103 29899081 PMC7143256

[B7] Badurdeen D. Hoff A. C. Barrichello S. Hedjoudje A. Itani M. I. Farha J. (2021). Efficacy of liraglutide to prevent weight regain after retrieval of an adjustable intra-gastric balloon—a case-matched study. *Obes. Surg.* 31 1204–1213. 10.1007/s11695-020-05117-8 33211267

[B8] Balsiger B. M. Poggio J. L. Mai J. Kelly K. A. Sarr M. G. (2000). Ten and more years after vertical banded gastroplasty as primary operation for morbid obesity. *J. Gastrointest. Surg.* 4 598–605. 10.1016/S1091-255X(00)80108-0 11307094

[B9] Barrichello S. Hourneaux, de Moura D. T. Hourneaux de Moura E. G. Jirapinyo P. Hoff A. C. (2019). Endoscopic sleeve gastroplasty in the management of overweight and obesity: an international multicenter study. *Gastrointest. Endosc.* 90 770–780. 10.1016/j.gie.2019.06.013 31228432

[B10] Bentham J. Di Cesare M. Bilano V. Bixby H. Zhou B. Stevens G. A. (2017). Worldwide trends in body-mass index, underweight, overweight, and obesity from 1975 to 2016: a pooled analysis of 2416 population-based measurement studies in 128⋅9 million children, adolescents, and adults. *Lancet* 390 2627–2642. 10.1016/S0140-6736(17)32129-3 29029897 PMC5735219

[B11] Billy H. T. Sarwer D. B. Ponce J. Ng-Mak D. S. Shi R. Cornell C. (2014). Quality of life after laparoscopic adjustable gastric banding (LAP-BAND): APEX interim 3-year analysis. *Postgrad. Med.* 126 131–140. 10.3810/pgm.2014.07.2791 25141251

[B12] Brolin R. E. Robertson L. B. Kenler H. A. Cody R. P. (1994). Weight loss and dietary intake after vertical banded gastroplasty and Roux-en-Y gastric bypass. *Ann. Surg.* 220 782–790. 10.1097/00000658-199412000-00012 7986146 PMC1234481

[B13] Bužga M. Machytka E. Klvaňa P. Kupka T. Zavadilová V. Zonča P. (2014). Effects of the intragastric balloon Medsil^®^ on weight loss, fat tissue, lipid metabolism, and hormones involved in energy balance. *Obes. Surg.* 24 909–915. 10.1007/s11695-014-1191-4 24488758 PMC4022986

[B14] Chandan S. Mohan B. P. Khan S. R. Facciorusso A. Ramai D. Kassab L. L. (2021). Efficacy and safety of intragastric balloon (IGB) in non-alcoholic fatty liver disease (NAFLD): a comprehensive review and meta-analysis. *Obes. Surg.* 31 1271–1279. 10.1007/s11695-020-05084-0 33409973

[B15] Chang P.-C. Chen K.-H. Huang I. Y.-W. Huang C.-K. Chen C.-Y. Wang M.-Y. (2021). Laparoscopic revision for gastric clipping: a single center experience and taiwan database review. *Obes. Surg.* 31 3653–3659. 10.1007/s11695-021-05466-y 33982242

[B16] Chang S.-H. Stoll C. R. T. Song J. Varela J. E. Eagon C. J. Colditz G. A. (2014). The effectiveness and risks of bariatric surgery: an updated systematic review and meta-analysis, 2003-2012. *JAMA Surg.* 149 275–287. 10.1001/jamasurg.2013.3654 24352617 PMC3962512

[B17] Chao S. H. Lin C. L. Lee W. J. Chen J. C. Chou J. J. (2019). Proximal jejunal bypass improves the outcome of gastric clip in patients with obesity and type 2 diabetes mellitus. *Obes. Surg.* 29 1148–1153. 10.1007/s11695-018-3607-z 30697678

[B18] De Jong K. Mathus-Vliegen E. M. H. Veldhuyzen E. A. M. L. Eshuis J. H. Fockens P. (2010). Short-term safety and efficacy of the trans-oral endoscopic restrictive implant system for the treatment of obesity. *Gastrointest. Endosc.* 72 497–504. 10.1016/j.gie.2010.02.053 20538274

[B19] de la Plaza Llamas R. Díaz Candelas D. A. Ramia J. M. (2020). Laparoscopic removal of a displaced vertical gastric clip causing gastric outlet obstruction. *Obes. Surg.* 30 2856–2857. 10.1007/s11695-020-04606-0 32314251

[B20] Doble B. Welbourn R. Carter N. Byrne J. Rogers C. A. Blazeby J. M. (2019). Multi-Centre micro-costing of Roux-En-Y gastric bypass, sleeve gastrectomy and adjustable gastric banding procedures for the treatment of severe, complex obesity. *Obes. Surg.* 29 474–484. 10.1007/s11695-018-3553-9 30368646

[B21] Edelman S. Ng-Mak D. S. Fusco M. Ashton D. Okerson T. Liu Q. (2014). Control of type 2 diabetes after 1 year of laparoscopic adjustable gastric banding in the helping evaluate reduction in obesity (HERO) study. *Diabetes, Obes. Metab.* 16 1009–1015. 10.1111/dom.12313 24824326

[B22] Favretti F. Ashton D. Busetto L. Segato G. De Luca M. (2009). The gastric band: first-choice procedure for obesity surgery. *World J. Surg.* 33 2039–2048. 10.1007/s00268-009-0091-6 19551427

[B23] FDA (2020). *UPDATE: Potential Risks with Liquid-filled Intragastric Balloons - Letter to Health Care Providers | FDA.* Available Online at: https://www.fda.gov/medical-devices/letters-health-care-providers/update-potential-risks-liquid-filled-intragastric-balloons-letter-health-care-providers-0 (accessed March 22, 2021).

[B24] Fittipaldi-Fernandez R. J. Zotarelli-Filho I. J. Diestel C. F. Klein M. R. S. T. de Santana M. F. de Lima J. H. F. (2020). Intragastric balloon: a retrospective evaluation of 5874 patients on tolerance, complications, and efficacy in different degrees of overweight. *Obes. Surg.* 30 4892–4898. 10.1007/s11695-020-04985-4 32959329

[B25] Flegal K. M. Carroll M. D. Kit B. K. Ogden C. L. (2012). Prevalence of obesity and trends in the distribution of body mass index among US adults, 1999-2010. *JAMA* 307 491–497. 10.1001/jama.2012.39 22253363

[B26] Froylich D. Abramovich T. S. Fuchs S. Zippel D. Hazzan D. (2020). Long-term (over 13 years) follow-up of vertical band gastroplasty. *Obes. Surg.* 30 1808–1813. 10.1007/s11695-020-04448-w 32048151

[B27] Fuller N. R. Lau N. S. Denyer G. Caterson I. D. (2013). An intragastric balloon produces large weight losses in the absence of a change in ghrelin or peptide YY. *Clin. Obes.* 3 172–179. 10.1111/cob.12030 25586733

[B28] Garb J. Welch G. Zagarins S. Kuhn J. Romanelli J. (2009). Bariatric surgery for the treatment of morbid obesity: a meta-analysis of weight loss outcomes for laparoscopic adjustable gastric banding and laparoscopic gastric bypass. *Obes. Surg.* 19 1447–1455. 10.1007/s11695-009-9927-2 19655209

[B29] Gasoyan H. Tajeu G. Halpern M. T. Sarwer D. B. (2019). Reasons for underutilization of bariatric surgery: the role of insurance benefit design. *Surg. Obes. Relat. Dis.* 15 146–151. 10.1016/j.soard.2018.10.005 30425002 PMC6441615

[B30] Gaździńska A. P. Mojkowska A. Zieliński P. Gazdzinski S. P. (2020). Changes in resting metabolic rate and body composition due to intragastric balloon therapy. *Surg. Obes. Relat. Dis.* 16 34–39. 10.1016/j.soard.2019.10.011 31734068

[B31] Genco A. Maselli R. Frangella F. Cipriano M. Paone E. Meuti V. (2013). Effect of consecutive intragastric balloon (BIB^®^) plus diet versus single BIB^®^ plus diet on eating disorders not otherwise specified (EDNOS) in obese patients. *Obes. Surg.* 23 2075–2079. 10.1007/s11695-013-1028-6 23881346

[B32] Golzarand M. Toolabi K. Farid R. (2017). The bariatric surgery and weight losing: a meta-analysis in the long- and very long-term effects of laparoscopic adjustable gastric banding, laparoscopic Roux-en-Y gastric bypass and laparoscopic sleeve gastrectomy on weight loss in adults. *Surg. Endosc.* 31 4331–4345. 10.1007/s00464-017-5505-1 28378086

[B33] Guo X. Mattar S. G. Mimms S. E. Navia J. A. Kassab G. S. (2014). Efficacy of a laparoscopic gastric restrictive device in an obese canine model. *Obes. Surg.* 24 159–166. 10.1007/s11695-013-1127-4 24214283

[B34] Guo X. Mattar S. G. Navia J. A. Kassab G. S. (2012). Response of gut hormones after implantation of a reversible gastric restrictive device in different animal models. *J. Surg. Res.* 178 165–171. 10.1016/j.jss.2012.02.032 22459287

[B35] Guo X. Zheng H. Mattar S. G. Lu X. Sandusky G. Navia J. A. (2011). Reversible gastric restriction implant: safety and efficacy in a canine model. *Obes. Surg.* 21 1444–1450. 10.1007/s11695-010-0299-4 21038085

[B36] Hindle A. Garcia X. D. P. Hayden M. O’Brien P. E. Brennan L. (2020). Pre-operative restraint and post-operative hunger, disinhibition and emotional eating predict weight loss at 2 Years post-laparoscopic adjustable gastric banding. *Obes. Surg.* 30 1347–1359. 10.1007/s11695-019-04274-9 32006239

[B37] Ibrahim A. M. Thumma J. R. Dimick J. B. (2017). Reoperation and medicare expenditures after laparoscopic gastric band surgery. *JAMA Surg.* 152 835–842. 10.1001/jamasurg.2017.1093 28514487 PMC5710463

[B38] Jacobs M. Zundel N. Plasencia G. Rodriguez-Pumarol P. Gomez E. Leithead J. (2017). A vertically placed clip for weight loss: a 39-month pilot study. *Obes. Surg.* 27 1174–1181. 10.1007/s11695-016-2432-5 27844255

[B39] Jain D. Bhandari B. S. Arora A. Singhal S. (2017). Endoscopic sleeve gastroplasty - a New tool to manage obesity. *Clin. Endosc.* 50 552–561. 10.5946/ce.2017.032 28607328 PMC5719914

[B40] Jensen M. D. Ryan D. H. Apovian C. M. Ard J. D. Comuzzie A. G. Donato K. A. (2014). 2013 AHA/ACC/TOS guideline for the management of overweight and obesity in adults: a report of the American college of cardiology/American heart association task force on practice guidelines and the obesity society. *J. Am. Coll. Cardiol.* 63 2985–3023. 10.1016/j.jacc.2013.11.004 24239920

[B41] Kawasaki T. Ohta M. Kawano Y. Masuda T. Gotoh K. Inomata M. (2015). Effects of sleeve gastrectomy and gastric banding on the hypothalamic feeding center in an obese rat model. *Surg. Today* 45 1560–1566. 10.1007/s00595-015-1135-1 25724939

[B42] Kellum J. M. Kuemmerle J. F. O’Dorisio T. M. Rayford P. Martin D. Engle K. (1990). Gastrointestinal hormone responses to meals before and after gastric bypass and vertical banded gastroplasty. *Ann. Surg.* 211 763–771. 10.1097/00000658-199006000-00016 2192696 PMC1358133

[B43] Kodner C. Hartman D. R. (2014). Complications of adjustable gastric banding surgery for obesity. *Am. Fam. Physician* 89 813–818. 24866217

[B44] Kumar N. (2015). Endoscopic therapy for weight loss: gastroplasty, duodenal sleeves, intragastric balloons, and aspiration. *World J. Gastrointest. Endosc.* 7 847–859. 10.4253/wjge.v7.i9.847 26240686 PMC4515419

[B45] Kumar N. Abu Dayyeh B. K. Lopez-Nava Breviere G. Galvao Neto M. P. Sahdala N. P. Shaikh S. N. (2018). Endoscopic sutured gastroplasty: procedure evolution from first-in-man cases through current technique. *Surg. Endosc.* 32 2159–2164. 10.1007/s00464-017-5869-2 29075966 PMC5845469

[B46] Kuzmak L. I. (1991). A review of seven years’ experience with silicone gastric banding. *Obes. Surg. Incl. Laparosc. Allied Care* 1 403–408. 10.1381/096089291765560809 10775942

[B47] Lee C. J. Florea L. Sears C. L. Maruthur N. Potter J. J. Schweitzer M. (2019). Changes in gut microbiome after bariatric surgery versus medical weight loss in a pilot randomized trial. *Obes. Surg.* 29 3239–3245. 10.1007/s11695-019-03976-4 31256356

[B48] Leonetti F. Silecchia G. Iacobellis G. Ribaudo M. C. Zappaterreno A. Tiberti C. (2003). Different plasma ghrelin levels after laparoscopic gastric bypass and adjustable gastric banding in morbid obese subjects. *J. Clin. Endocrinol. Metab.* 88 4227–4231. 10.1210/jc.2003-030133 12970291

[B49] Lopez-Nava G. Galvao M. Bautista-Castaño I. Fernandez-Corbelle J. Trell M. (2016). Endoscopic sleeve gastroplasty with 1-year follow-up: factors predictive of success. *Endosc. Int. Open* 04 E222–E227. 10.1055/s-0041-110771 26878054 PMC4751018

[B50] Lopez-Nava G. Negi A. Bautista-Castaño I. Rubio M. A. Asokkumar R. (2020). Gut and metabolic hormones changes after endoscopic sleeve gastroplasty (ESG) vs. laparoscopic sleeve gastrectomy (LSG). *Obes. Surg.* 30 2642–2651. 10.1007/s11695-020-04541-0 32193741

[B51] Lopez-Nava G. Sharaiha R. Z. Vargas E. J. Bazerbachi F. Manoel G. N. Bautista-Castaño I. (2017). Endoscopic sleeve gastroplasty for obesity: a multicenter study of 248 patients with 24 months follow-up. *Obes. Surg.* 27 2649–2655. 10.1007/s11695-017-2693-7 28451929

[B52] Madsbad S. Dirksen C. Holst J. J. (2014). Mechanisms of changes in glucose metabolism and bodyweight after bariatric surgery. lancet. *Diabetes Endocrinol.* 2 152–164. 10.1016/S2213-8587(13)70218-3 24622719

[B53] Mason E. E. (1982). Vertical banded gastroplasty for obesity. *Arch. Surg.* 117 701–706. 10.1001/archsurg.1982.01380290147026 7073493

[B54] Mathus-Vliegen E. M. H. de Groot G. H. (2013). Fasting and meal-induced CCK and PP secretion following intragastric balloon treatment for obesity. *Obes. Surg.* 23 622–633. 10.1007/s11695-012-0834-6 23224567

[B55] Mathus-Vliegen E. M. H. Eichenberger R. I. (2014). Fasting and meal-suppressed ghrelin levels before and after intragastric balloons and balloon-induced weight loss. *Obes. Surg.* 24 85–94. 10.1007/s11695-013-1053-5 23918282

[B56] Mion F. Napoléon B. Roman S. Malvoisin E. Trepo F. Pujol B. (2005). Effects of intragastric balloon on gastric emptying and plasma ghrelin levels in non-morbid obese patients. *Obes. Surg.* 15 510–516. 10.1381/0960892053723411 15946431

[B57] Monteiro M. P. Ribeiro A. H. Nunes A. F. Sousa M. M. Monteiro J. D. Águas A. P. (2007). Increase in ghrelin levels after weight loss in obese Zucker rats is prevented by gastric banding. *Obes. Surg.* 17 1599–1607. 10.1007/s11695-007-9324-7 18049841

[B58] Noel P. Eddbali I. Nedelcu M. (2020). Laparoscopic clip gastroplasty with the BariClip. *Obes. Surg.* 30 5182–5183. 10.1007/s11695-020-04803-x 32996101

[B59] Noel P. Nedelcu A. M. Eddbali I. Zundel N. (2018). Laparoscopic vertical clip gastroplasty - quality of life. *Surg. Obes. Relat. Dis.* 14 1587–1593. 10.1016/j.soard.2018.07.013 30449515

[B60] Novikov A. A. Afaneh C. Saumoy M. Parra V. Shukla A. Dakin G. F. (2018). Endoscopic sleeve gastroplasty, laparoscopic sleeve gastrectomy, and laparoscopic band for weight loss: how do they compare? *J. Gastrointest. Surg.* 22 267–273. 10.1007/s11605-017-3615-7 29110192

[B61] Ogden C. L. Carroll M. D. Lawman H. G. Fryar C. D. Kruszon-Moran D. Kit B. K. (2016). Trends in obesity prevalence among children and adolescents in the United States, 1988-1994 through 2013-2014. *JAMA* 315 2292–2299. 10.1001/jama.2016.6361 27272581 PMC6361521

[B62] Olbers T. Björkman S. Lindroos A. Maleckas A. Lönn L. Sjöström L. (2006). Body composition, dietary intake, and energy expenditure after laparoscopic Roux-en-Y gastric bypass and laparoscopic vertical banded gastroplasty: a randomized clinical trial. *Ann. Surg.* 244 715–722. 10.1097/01.sla.0000218085.25902.f8 17060764 PMC1856598

[B63] Opozda M. Chur-Hansen A. Wittert G. (2016). Changes in problematic and disordered eating after gastric bypass, adjustable gastric banding and vertical sleeve gastrectomy: a systematic review of pre-post studies. *Obes. Rev.* 17 770–792. 10.1111/obr.12425 27296934

[B64] Park C. H. Nam S.-J. Choi H. S. Kim K. O. Kim D. H. Kim J.-W. (2019). Comparative efficacy of bariatric surgery in the treatment of morbid obesity and diabetes mellitus: a systematic review and network meta-analysis. *Obes. Surg.* 29 2180–2190. 10.1007/s11695-019-03831-6 31037599

[B65] Paulus G. F. van Avesaat M. Crijnen J. A. W. Ernest, van Heurn L. W. Westerterp-Plantenga M. S. (2020). Preliminary evidence that endoscopic gastroplication reduces food reward. *Appetite* 150:104632. 10.1016/j.appet.2020.104632 32070711

[B66] Ponce J. Taheri S. Lusco V. Cornell C. Ng-Mak D. S. Shi R. (2014). Efficacy and safety of the adjustable gastric band-pooled interim analysis of the APEX and HERO studies at 48 weeks. *Curr. Med. Res. Opin.* 30 841–848. 10.1185/03007995.2013.874992 24328415

[B67] Pontiroli A. E. Zakaria A. S. Fanchini M. Osio C. Tagliabue E. Micheletto G. (2018). A 23-year study of mortality and development of co-morbidities in patients with obesity undergoing bariatric surgery (laparoscopic gastric banding) in comparison with medical treatment of obesity. *Cardiovasc. Diabetol.* 17:161. 10.1186/s12933-018-0801-1 30594184 PMC6311074

[B68] Popov V. B. Ou A. Schulman A. R. Thompson C. C. (2017). The impact of intragastric balloons on obesity-related co-morbidities: a systematic review and meta-analysis. *Am. J. Gastroenterol.* 112 429–439. 10.1038/ajg.2016.530 28117361

[B69] Pournaras D. J. Osborne A. Hawkins S. C. Vincent R. P. Mahon D. Ewings P. (2010). Remission of type 2 diabetes after gastric bypass and banding: mechanisms and 2 year outcomes. *Ann. Surg.* 252 966–971. 10.1097/SLA.0b013e3181efc49a 21107106

[B70] Ramai D. Singh J. Mohan B. P. Madedor O. Brooks O. W. Barakat M. (2020). Influence of the elipse intragastric balloon on obesity and metabolic profile: a systematic review and meta-analysis. *J. Clin. Gastroenterol.* online ahead of print. 10.1097/MCG.0000000000001484 33394629

[B71] Schlager A. Khalaileh A. Mintz Y. Gazala M. A. Globerman A. Ilani N. (2011). A mouse model for sleeve gastrectomy: applications for diabetes research. *Microsurgery* 31 66–71. 10.1002/micr.20797 20734435

[B72] Sharaiha R. Z. Kumta N. A. Saumoy M. Desai A. P. Sarkisian A. M. Benevenuto A. (2017). Endoscopic sleeve gastroplasty significantly reduces body mass index and metabolic complications in obese patients. *Clin. Gastroenterol. Hepatol.* 15 504–510. 10.1016/j.cgh.2016.12.012 28017845

[B73] Smith K. E. Orcutt M. Steffen K. J. Crosby R. D. Cao L. Garcia L. (2019). Loss of control eating and binge eating in the 7 years following bariatric surgery. *Obes. Surg.* 29 1773–1780. 10.1007/s11695-019-03791-x 30820886 PMC6948918

[B74] Sonavane S. K. Menias C. O. Kantawala K. P. Shanbhogue A. K. Prasad S. R. Eagon J. C. (2012). Laparoscopic adjustable gastric banding: what radiologists need to know. *Radiographics* 32 1161–1178. 10.1148/rg.324115177 22787000

[B75] Sysko R. Devlin M. J. Schebendach J. Tanofsky-Kraff M. Zimmerli E. Korner J. (2013). Hormonal responses and test meal intake among obese teenagers before and after laparoscopic adjustable gastric banding. *Am. J. Clin. Nutr.* 98 1151–1161. 10.3945/ajcn.113.061762 23985807 PMC3798074

[B76] Tremaroli V. Karlsson F. Werling M. Ståhlman M. Kovatcheva-Datchary P. Olbers T. (2015). Roux-en-Y gastric bypass and vertical banded gastroplasty induce long-Term changes on the human gut microbiome contributing to fat mass regulation. *Cell Metab.* 22 228–238. 10.1016/j.cmet.2015.07.009 26244932 PMC4537510

[B77] Tsai C. Zehetner J. Beel J. Steffen R. (2019). Long-term outcomes and frequency of reoperative bariatric surgery beyond 15 years after gastric banding: a high band failure rate with safe revisions. *Surg. Obes. Relat. Dis.* 15 900–907. 10.1016/j.soard.2019.03.017 31378280

[B78] van der Wielen N. Paulus G. van Avesaat M. Masclee A. Meijerink J. Bouvy N. (2017). Effect of endoscopic gastroplication on the genome-wide transcriptome in the upper gastrointestinal tract. *Obes. Surg.* 27 740–748. 10.1007/s11695-016-2356-0 27620343 PMC5306242

[B79] van Wezenbeek M. R. Smulders J. F. de Zoete J. P. J. G. M. Luyer M. D. van Montfort G. Nienhuijs S. W. (2015). Long-term results of primary vertical banded gastroplasty. *Obes. Surg.* 25 1425–1430. 10.1007/s11695-014-1543-0 25519773

[B80] Vargas E. J. Rizk M. Bazerbachi F. Abu Dayyeh B. K. (2018). Medical devices for obesity treatment: endoscopic bariatric therapies. *Med. Clin. North Am.* 102 149–163. 10.1016/j.mcna.2017.08.013 29156183

[B81] Verlaan T. de Jong K. de la Mar-Ploem E. D. Veldhuyzen E. A. Mathus-Vliegen E. M. Fockens P. (2016). Trans-oral endoscopic restrictive implant system: endoscopic treatment of obesity? *Surg. Obes. Relat. Dis.* 12 1711–1718. 10.1016/j.soard.2016.02.027 27317594

[B82] Vyas D. Deshpande K. Pandya Y. (2017). Advances in endoscopic balloon therapy for weight loss and its limitations. *World J. Gastroenterol.* 23 7813–7817. 10.3748/wjg.v23.i44.7813 29209122 PMC5703910

[B83] Wang Y. Guo X. Lu X. Mattar S. Kassab G. (2019). Mechanisms of weight loss after sleeve gastrectomy and adjustable gastric banding: far more than just restriction. *Obesity* 27 1776–1783. 10.1002/oby.22623 31545007

[B84] Wei J.-H. Yeh C.-H. Lee W.-J. Lin S.-J. Huang P.-H. (2020). Sleeve gastrectomy in mice using surgical clips. *J. Vis. Exp.*, e60719. 10.3791/60719 33252113

[B85] Wharton S. Lau D. C. W. Vallis E. M. Sharma A. M. Biertho L. Campbell-Scherer D. (2020). Obesity in adults: a clinical practice guideline. *CMAJ* 192 E875–E891. 10.1503/cmaj.191707 32753461 PMC7828878

[B86] Yumuk V. Tsigos C. Fried M. Schindler K. Busetto L. Micic D. (2015). European guidelines for obesity management in adults. *Obes. Facts* 8 402–424. 10.1159/000442721 26641646 PMC5644856

